# Eroded adjustable gastric band migration causing gastric obstruction and perforation in a pregnant lady

**DOI:** 10.1016/j.ijscr.2020.04.087

**Published:** 2020-05-19

**Authors:** Maram Alawad, Muhammad Abukhater, Khalid Al-Mohaimeed

**Affiliations:** Department of Surgery, P.O Box 59046, King Fahad Medical City, Riyadh 11525, Saudi Arabia

**Keywords:** Gastric band, Bariatric surgery, Pregnancy, Complications, Case report, Gastro-gastrostomy

## Abstract

**Background:**

The laparoscopic adjustable gastric band is the least invasive bariatric surgery, which has the advantage of preserving the anatomy of the gastrointestinal tract. In the last two decades, the laparoscopic adjustable gastric band (LAGB) has largely been replaced by other surgical techniques for weight loss because of its high inseparable complications. Although a popular surgical technique at the time of its introduction in 1993, LAGB nowadays reports for not more than 5.5% of all bariatric procedures. The estimated overall long term complication rates of LAGB are ranging from 0.1% to 28%.

**Objective:**

In this report, we review one of the cases that are considered as an anecdotic event that might follow one of the bariatric procedures.

**Methods:**

We reported a pregnant lady who undergoes gastric banding 12 years ago. presented with progressively increasing colicky epigastric pain, nausea, and vomiting. The investigations show gastric obstruction and perforation secondary to eroded gastric band and migration.

**Results:**

The management was completed by the elimination of the gastric band proceed with central gastrectomy plus gastro-gastrostomy with an excellent outcome for the patient and her baby.

**Conclusion:**

This case highlights an absolutely rare serial complication, that may happen even when the patient presents with a vague complaint. Intraoperative findings can determine the way of management to achieve suitable results. Lastly; we encourage the young Women who underwent or planning for bariatric surgery to do a careful follow-up.

## Highlights

• Laparoscopic adjustable gastric banding (LAGB) was considered as one of the most effective management for morbid obesity, with outstanding long-term results on weight loss, comorbidities, and quality of life.
• Reported complications of LAGB include erosion, infection, migration, obstruction, and rarely ischemia.
• We report a case of pregnant woman who underwent LAGB 12 years ago diagnosed as gastric obstruction and perforation that was managed by central gastrectomy with gastro-gastrostomy.
• We encourage the bariatric surgeons to be attentive LAGB complications among women planning for pregnancy.

## 1 Background

The laparoscopic adjustable gastric band is the least invasive bariatric surgery, which has the advantage of preserving the anatomy of the gastrointestinal tract [1]. In the last two decades, the laparoscopic adjustable gastric band (LAGB) has largely been replaced by other surgical techniques for weight loss because of its high inseparable complications. Although a popular surgical technique at the time of its introduction in 1993, LAGB nowadays reports for not more than 5.5% of all bariatric procedures [2]. The estimated overall long term complication rates of LAGB are ranging from 0.1% to 28% [3,4]. The work has been reported in line with the SCARE criteria [22].

## 2 Case presentation

A 39 *primigravida* pregnant woman at 8 weeks of gestational age through IVF pregnancy. Status post LAGB in 2006 that was operated in another hospital for morbid obesity with a baseline body weight 108 kg (BMI: 50.7 kg/m^2^). She experienced weight loss of 62 kg (BMI: 21.5 kg/m^2^) after the band applied, which she had maintained for the previous 12 years with the last gastric band adjustment performed 3 years back without apparent difficulties. Thenceforth and for unknown reasons, the follow-up ceased. She remained without obvious complications and regained weight up to a 62 kg (BMI of 29.1 kg/m^2^) until her current presentation. The patient presented to emergency department complaining of progressively increasing colicky abdominal pain concentrated in intensity in the epigastrium since one week, associated with nausea and emesis. No history of fever, change in bowel habit or shortness of breath. Also, the patient denies any previous hospital admissions with similar symptoms, except the upper gastrointestinal endoscopy done in private hospital two days prior to emergency department presentation for abdominal discomfort and she was discharged without any intervention. She is not known to have any chronic illness or previous surgery apart from the gastric band. Physical examination revealed mild discomfort at the previous site of laparoscopic gastric band port in the epigastrium exacerbated by movement. Vital signs records were normal.

## 3 Investigations

**Fig. 1:**
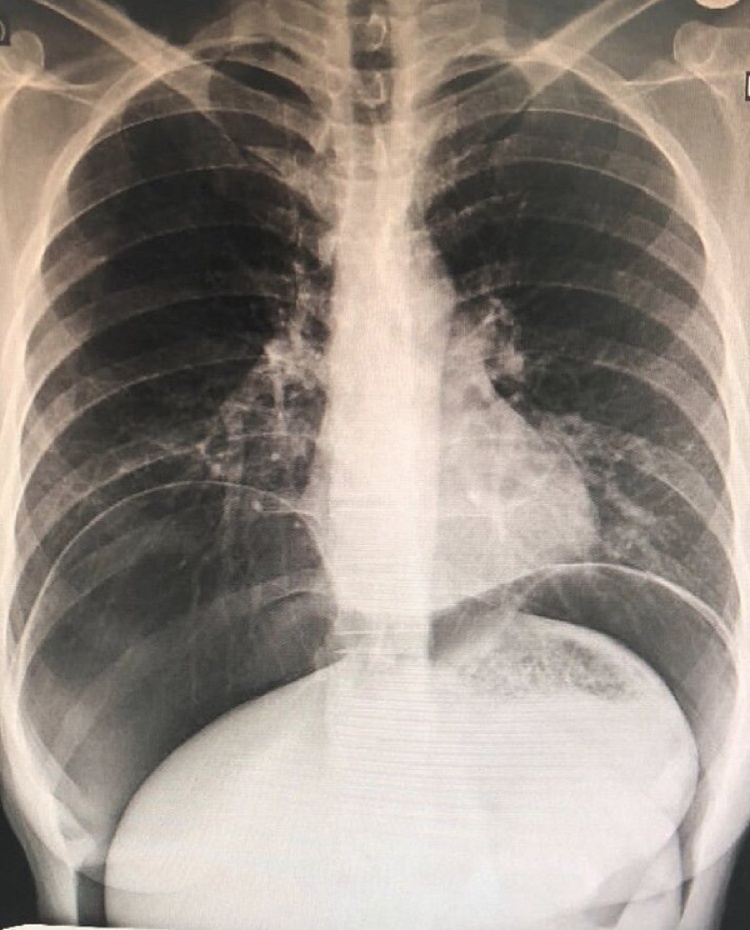
Chest x-ray showed significant bilateral pneumoperitoneum with notice of the gastric band.

**Fig. 2:**
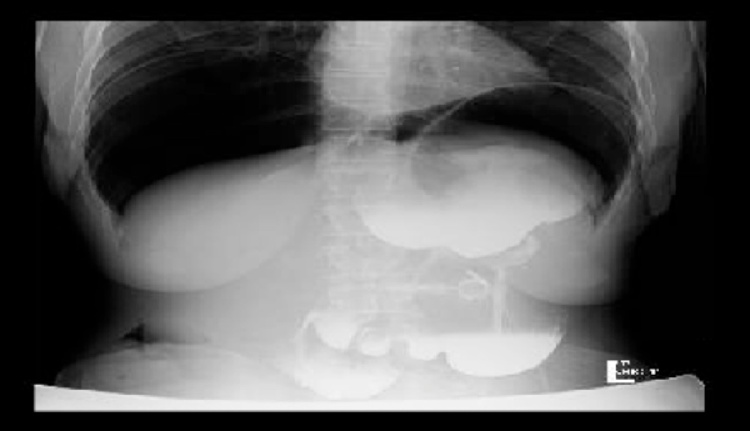
X-ray abdomen (one shout) erect view with oral gastrografin outlined the stomach and duodenum with huge dilated funds and proximal body of stomach proximally to the site of a gastric band which is displaced downward, with no leak.

**Fig. 3:**
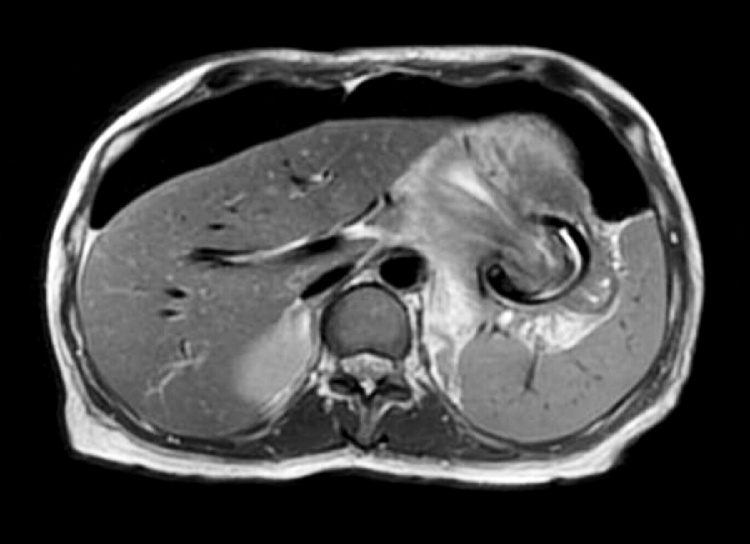
A subsequent Multi Sequential Multiplanar (MRI) of abdomen confirmed gastric band migrated inferiorly causing dilating of stomach funds with small erosions.

**Fig. 4:**
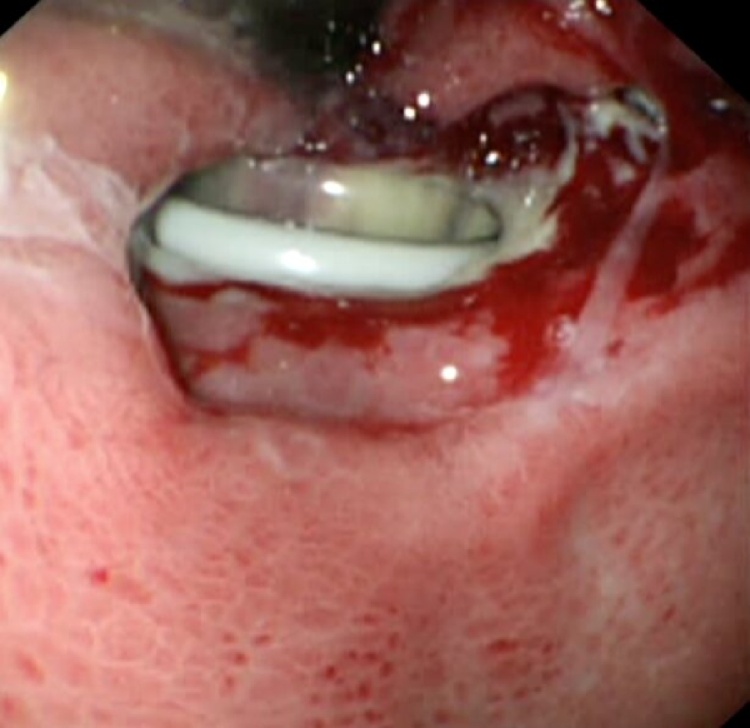
The upper gastrointestinal endoscopy done in private hospital showed completely eroded band through the gastric wall, located almost intraluminal. Also, the scope could not pass beyond the band Zone.

Biochemical and hematological investigations were normal within normal limits. Chest x-ray showed significant bilateral pneumoperitoneum with notice of the gastric band (Fig. 1). The patient was admitted, kept NPO on IVF. As the patient was pregnant, an x-ray abdomen (one shout) erect view with oral gastrografin was taken. The x-ray abdomen outlined the stomach and duodenum with huge dilated funds and proximal body of stomach proximally to the site of a gastric band which is displaced downward, with no leak (Fig. 2). A subsequent Multi Sequential Multiplanar (MRI) of abdomen confirmed gastric band migrated inferiorly causing dilating of stomach funds with small erosions. No gross perforation or collection. Large pneumoperitoneum was noted (Fig. 3). The upper gastrointestinal endoscopy done in private hospital showed completely eroded band through the gastric wall, located almost intraluminal. Also, the scope could not pass beyond the band Zone (Fig. 4).

## 4 Differential diagnosis

The leading diagnosis after evaluating the MRI of abdomen was gastric erosion and obstruction with perforation secondary to laparoscopic gastric band. However, other differential diagnoses included cholecystitis, gastroenteritis and gastrointestinal perforation as well as actopic pregnency was consider.

## 5 Treatment

**Fig. 5:**
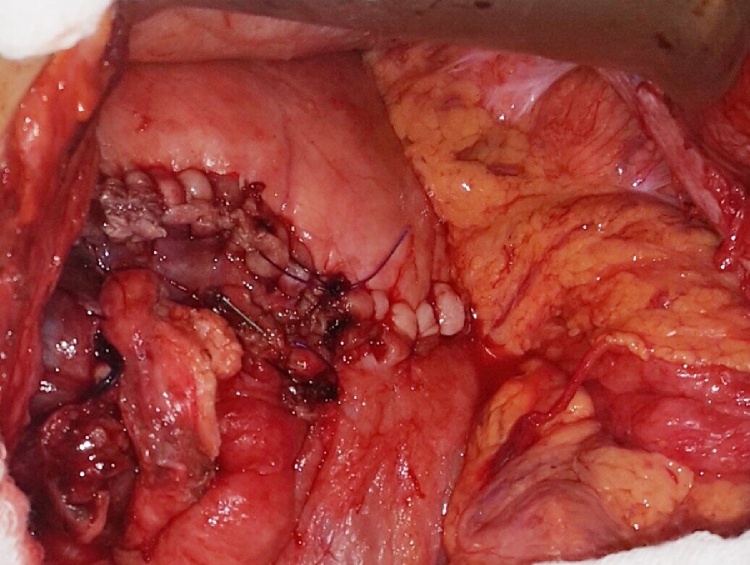
Intraoperative picture showing (hand swing) gastro gastrostomy.

**Fig. 6:**
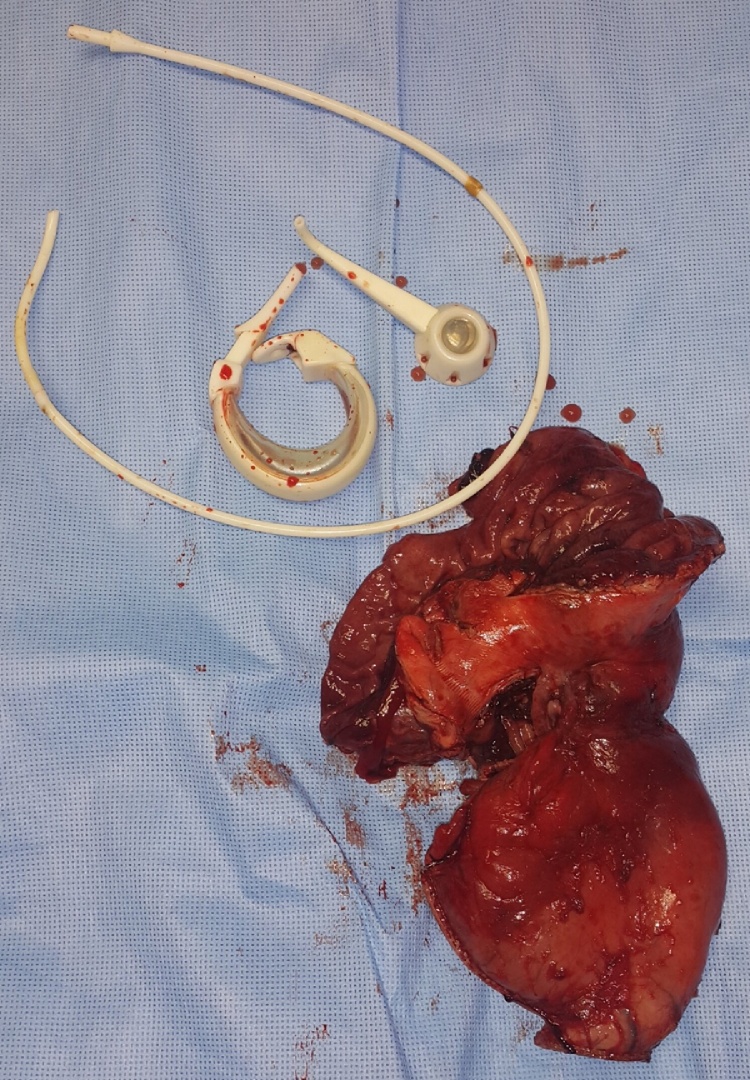
Pathology specimen.

Initially, the patient was taken to the operation room underwent diagnostic laparoscopy. The laparoscopy explored huge stomach dilatation proximal to the band with fibrosis and adhesion all-over the band area. For the patient safety, since she was pregnant, the decision was made to convert operation to open surgery, which was started by midline laparotomy. Afterward, adhesion was released and the abdomen was explored. Migration of the band was seen to mid stomach body that made fibrosis ring and massive fundus and body dilatation proximally, in addition to an extremely thickened stomach wall. Accordingly, the band was removed and found to be completely obstructing the lumen and eroded posteriorly to the pancreas. We made a mobilization of the posterior aspect of the stomach and release adhesion of the antrum with manual pyloric dilatation. Subsequently, central gastrectomy plus gastro gastrostomy (hand swing) was done (Fig. 5). With no immediate intraoperative complication. The specimen (Fig. 6) sends to the pathology lab shows a feature of chronic inflammation with no malignancy.

## 6 Outcome and follow-up

Postoperative was uneventful, so the IV antibiotic and IVF was stopped and the diet was advanced. When the patient leaving the hospital and during the follow-ups was in good condition and after a few months gave birth to a healthy child.

## 7 Discussion

In this case report, we present a challenging condition for a pregnant lady who complained of acute abdominal pain diagnosed with a combination of four gastric band complications including band erosion and, band migration through the gastric lumen that leads to obstruction and perforation. Among these complications, acute gastric ischemia with associated perforation has been only reported rarely [5]. Although, the gastric band erosion with intragastric band migration consider the most worrisome complaction because of the probability of consequent obstruction, peritonitis, and sepsis [6]. Gastric erosion and intragastric band migration may appear either in the early or in the late postoperative settings. Pathogenesis of gastric band erosion is multifactorial and is dependent on the postoperative time frame. and what is has been mentioned in the literature regarding Late band erosions, its typically occur at least 24 months after surgery and are favored to be secondary to sequela of a pressure-induced response or a foreign body rejection reaction [7]. The majority of patient with Gastric band complications usually asymptomatic and it commonly discovered during routine screening with gastroscopy [8]. Furthermore There are no specific symptoms that predict gastric band complications, so Symptomatic patients may present with vague abdominal pain, weight gain resulting to loss of band function, hematemesis, infection, transient or complete obstruction, and rarely bowel perforation [9]. The radiographic and fluoroscopic studies is required during the assessment to confirm the definitive diagnosis, in early sittings it might be Uncertain in absence of previous imaging studies for comparison [10]. In later stages, oral contrast material may be identified surrounding the portion of the band that has eroded into the gastric lumen and increasing accuracy of CT imaging by distended the stomach and by thorough review of multiplanar images [18]. In uncertain cases, a definitive diagnosis can be made with endoscopic examination [8]. In this case, a simple chest x-ray was performed in accordance with the guidelines for pregnant women [10]. In addition, we performed an x-ray abdomen (one shout) erect view with oral gastrografin to provide high-quality imaging to make a definitive medical diagnosis. A fetal radiation doses of less than 50 mGy are not associated with increased fetal anomalies or fetal loss throughout pregnancy [11]. Himpens and his colleagues reported that 50% of band removal was for complications in a cohort with 13 years’ median follow-up [12]. In another study reported that 42% of bands were removed with or without bypass conversion in a cohort with a mean follow-up of 14 years [13]. The majority of late complications after LABG that require surgery (to reposition, remove, or replace the band) can be managed laparoscopically. However, in cases of gastric necrosis or perforation, laparotomy has been proposed as necessary, and the use of laparoscopy has been anecdotal [14]. In the case presented, urgent band removal was imperative. Endoscopic removal techniques have been described in the literature, regardless of whether the band is contained completely within the gastric lumen [15,16]. Because endoscopic retreating of the band was unattainable, the surgical methods were indicated. In our patient during the operation, As the intraoperative finding was described, including hugely dilated of the proximal part of the stomach to the band and incredibly thickening of the stomach wall, Roux-en-Y surgery which is considered the stander operation in this kind of complications was not preferable. through literature many surgical methods have been described primarily depended on the situation interpretively, healthful lesser curvature could allow gastric tubulization, which guided us to perform a laparoscopic sleeve gastrectomy [17,18]. In addition, if the greater curvature is not viable, a possible alternative may be considering a subtotal gastrectomy or a gastric bypass. If the cardia is not viable, total gastrectomy with esophagojejunostomy may be compulsory. Conversely, in cases in which the lesser or greater curvatures is completely preserved, sleeve gastrectomy may be the best option; it avoids the need for gastrointestinal anastomosis, and its related consequences [17,18]. In this case the decision was made to proceed by central gastrectomy with gastrogastrostomy, As with the gastric perforation at the migrated band in the body of the stomach beside huge dilatation above the slipped band combined and contamination of the area, the first option was to do gastric bypass above the perforation area. But due to chronic obstruction, the wall of the stomach was very thick so they will discrepancy with small bowel and the anastomosis will leak, For this reason, we preferred to do central gastrectomy, so, the suture will hold better. Furthermore, this method was carried out effectively as an alternative management for gastric ulcers in an attempt to avoid postoperative dumping syndrome and bile reflux, complaints that were repeatedly seen after conventional distal gastrectomy with a Billroth-I anastomosis [19,20] as well as intraoperative manual dilatation of pylorus, which provide prevention from pyloric stenosis caused by pyloric spasms [21].

## 8 Conclusion

This case highlights an absolutely rare serial complication. Intraoperative findings could determine the way of management to achieve suitable results. Furthermore ; The possibility of serious complications still there in patient came with a vague complain.

## Funding

None.

## Ethical approval

This study was approved by the Institutional Review Board of our institution (approval 18-604).

## Consent

Written informed consent was obtained from the patient for publication of this case report and accompanying images. A copy of the written consent is available for review by the Editor-in-Chief of this journal on request.

## Author contribution

Dr. Maram Alawad: Chosen the design of study, writing the paper. Dr. Muhammad Abukhatar: Patient was admitted and treated under care of Dr. Abukhatar and Dr. Almohimeed, they were help in collect, analyse and interpreted the data. Dr. Khalid Al-Mohaimeed: Patient was admitted and treated under Care of Dr. Abukhater and Dr. Almohimeed, they were help in collects, analyse and interpreted the data.

## Registration of research studies

This study was approved by the Institutional Review Board of our institution (approval 18-604).

## Guarantor

Dr. Maram Alawad.

## Provenance and peer review

Not commissioned, externally peer-reviewed.

## Declaration of Competing Interest

None.
